# Measurement of Workability of Fresh Concrete Using a Mixing Truck

**DOI:** 10.6028/jres.110.006

**Published:** 2005-02-01

**Authors:** Sofiane Amziane, Chiara F. Ferraris, Eric P. Koehler

**Affiliations:** Laboratoire de Génie Mécanique, et Matériaux, Université de, Bretagne Sud, rue St Maudé, BP, 92116-56321 Lorient Cedex-France; National Institute of Standards and Technology, Gaithersburg, MD 20899-0001, USA; University of Texas at Austin, Austin, TX, USA

**Keywords:** concrete, concrete rheometer, mixer truck, rheology

## Abstract

The main objective of this study is to evaluate the workability of fresh portland cement concrete while it is still in the mixing truck by determining fundamental rheological parameters (plastic viscosity and yield stress). Nine concrete mixtures with different values of yield stress and plastic viscosity were tested in a concrete truck. The measurements made with the truck were based on the typical method of determining the flow behavior in a traditional fluid rheometer; that is, the shear rate in the mixing truck was swept from high to low by varying the rotation speed of the drum. The results of these experiments are discussed and compared with data provided by the ICAR rheometer, a portable rheometer designed for measuring concrete rheology. The test results indicate that the mixing truck equipment is sufficiently sensitive to detect differences in yield stress, slump, and plastic viscosity. However, the plastic viscosity determined by the truck measurement did not correlate with plastic viscosity as measured by the ICAR rheometer, while the yield stress determined by the truck measurement did correlate well with the measured slump and the ICAR rheometer results

Suggestions are given on how to improve the mixing truck for better use as a rheometer.

## 1. Introduction

The use of highly fluid concretes, the rheology of which cannot be suitably characterized with the slump test (ASTM C 143 [1]) alone, has resulted in the emergence of many new methods for characterizing the flow of freshly mixed concrete. A synthesis of more than 61 existing test methods [2] for workability characterization found that available devices vary widely in their geometry, cost, method of operation, and suitability for field use.

To describe concrete flow behavior, both yield stress and plastic viscosity, as defined by the Bingham model, are key properties that should be determined. The measurement of these parameters is currently possible only by using a rheometer adapted to concrete [3,4,5]. Unfortunately, the current cost of these devices (even if very low compared to the cost of the produced concrete) and their complexities, as compared to the slump test, has restricted the use of rheological measurements mainly to research laboratories.

The idea of measuring rheological properties during mixing is not new [6]. In fact, some existing concrete rheometers are based on this idea. These devices operate by measuring the torque induced on a mixing blade rotated at a range of different speeds. Another possibility for measuring concrete flow properties is based on relating the energy data recorded during mixing, as investigated by de Larrard et al. [7,8]. These researchers compared the curve of electric power versus mixing time of the concrete with the measurements obtained from their rheometer and were able to provide a correlation curve between the two instruments [7,8].

The scatter in data from one rheometer to another can reduce the trust an operator has in a given measurement [3,4,5]. Indeed, the slump test has remained the standard tool throughout the world for characterizing the workability of freshly mixed concrete because of the device’s simple calibration, which creates little ambiguity or confusion. The slump test, however, only measures a value related to yield stress, which is insufficient for fully describing the flow properties of concrete. An attempt to modify the slump test to measure plastic viscosity showed the limits of such an approach [9]. A simple and reliable method of rheological characterization adapted to the needs of industry is needed. In particular, few studies exist on the prospect of determining the flow properties of concrete in a mixing truck in transit to a jobsite. To the authors’ knowledge, only one study has been published on this topic [10].

An analysis of the concrete production process shows that the transport phase in a mixing truck—particularly just before discharging concrete from the truck—is the most suitable time to measure rheological properties. In order to make rheological measurements during the mixing process, the mixing truck must be able to mix the concrete at different speeds to generate a range of shear rates. A mixer in a central plant—despite being considered more efficient than a truck mixer—is not typically capable of operating at different speeds [6,11]. Given this background, an investigation of the feasibility of determining rheological properties by using the available data from a mixing truck was conducted.

## 2. Experimental Program

### 2.1 Materials^1^

The cementitious materials used in all mixtures consisted of an ASTM C 150 Type I portland cement with a Blaine specific surface of 467 m^2^/kg and a density of 3050 kg/m^3^ and a ground granulated blast furnace slag. The two aggregates were a natural sand denoted “*La Plata*” and a coarse aggregate denoted “*Brandywine*”. The sand had a maximum size of 3 mm and consisted of a mass fraction of 2.8 % of particles with dimensions smaller than 0.15 mm.

All admixtures used were commercially available products. All concrete mixtures incorporated a water-reducing and retarding admixture based on a sodium salt of organic acid mixture and with a specific gravity of 1.2. The high-range water-reducing admixture (HRWRA) was a polycarboxylate-based admixture with a specific gravity of 1.1. A viscosity modifying admixture was also used.

### 2.2 Mixture Proportions

Two distinct control mixtures of concrete—with the same types of materials but with different proportions—were used in the experimental program. As summarized in Table 1, the two control mixtures were denoted C10 and C20. These control mixtures were subsequently modified by using a high-range water-reducing admixture to increase slump (C11, C12, C13, C21, C22), incorporating a viscosity-modifying admixture (VMA) to increase viscosity (C14), or adding water to increase slump (C23). A total of nine concrete mixtures were tested in this study.

### 2.3 Rheometers

#### 2.3.1 Using the Mixing Truck as a Rheometer

To transform a truck mixer into a rheometer requires that at least two entities be measured: the rotational speed of the drum and the power consumption or torque used by the mixer motor during rotation. To obtain both the yield stress and the viscosity it is necessary to obtain data at several speeds. The methodology proposed here requires the measurement of the power during mixing, the load volume, the mass of concrete, and the shear rate in the concrete, which is deduced from the drum rotational speed and geometrical characteristics.

**Fig. 1:**
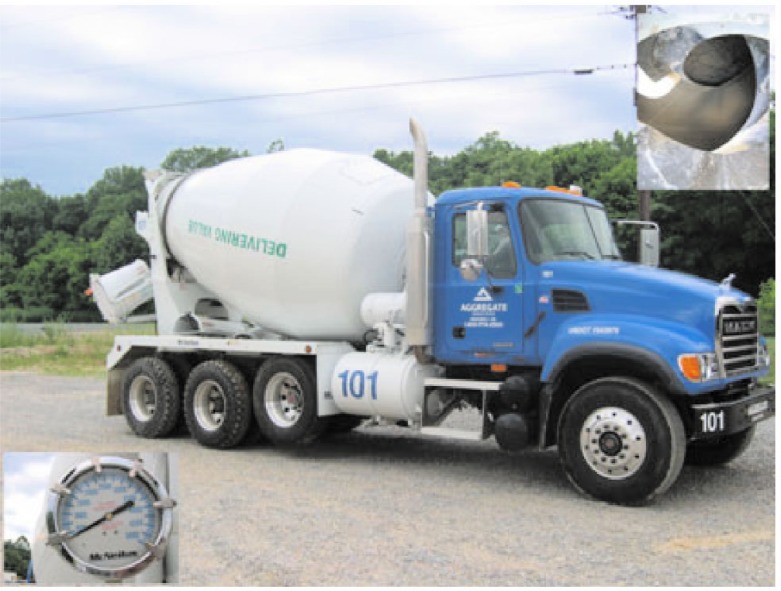
View of the used truck, the slump indicator (bottom left) and the interior of the drum (top right).

The values of these two variables (power and shear rate) at different speeds may be plotted against each other. The slope of this resulting curve according to the Bingham model will give the plastic viscosity and the intercept at zero shear rate will give the yield stress. The concrete truck mixer used (Fig. 1) was fitted with a device capable of measuring the oil pressure to turn the drum (also called slump meter). The drum speed measurements were manually made by two persons using a stopwatch. The Bingham test involves sweeping shear rates from high to low and measuring the stress at various shear rates. Therefore, the drum was turned at the highest possible speed, 1.74 rad/s (16.66 rpm), and then gradually decreased in discrete steps to zero while the oil pressure was measured.

**Fig. 2:**
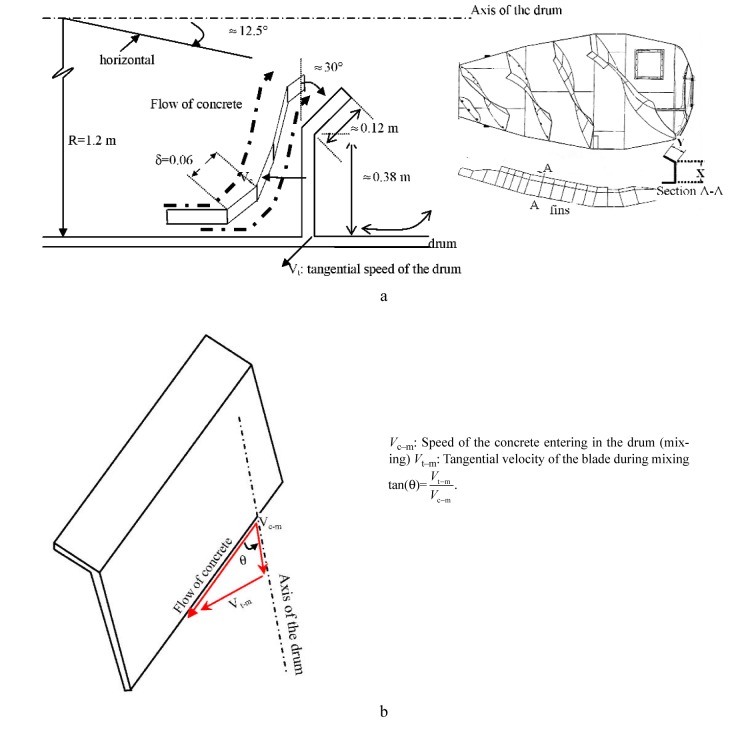
Geometrical characteristics of the drum of the used mixing truck. (a) Flow of plastic concrete relative to blades during mixing. The concrete flow was modelled as continuous discrete finite elements. The square elements have *δ* = 0.06 m sides. The higher shear rate is certainly localized at the angled end of the blade. (b) details of flow of concrete around one of the blades.

The calculation method to determine the shear rate in the drum from the speed and the truck geometrical characteristics was developed in Ref. [12]. The truck used had a capacity of about 7.5 m^3^, with a drum radius, *R*, of 1.20 m. The maximum drum speed, *n*, was 1.74 rad/s (16.66 rpm or 0.278 rps). These data were provided without uncertainty information by the manufacturer of the truck. The tangential velocity of concrete in the drum during mixing (Fig. 2b) can be calculated using Eq. (1):

$$V_{t-m}=R\cdot n=1.20\cdot 1.74=2.1\cdot m/s$$ (1)

with *n* in rad/s and *R* in m.

The truck drum used is inclined at a small angle^2^, 12.5°, to allow the concrete to slide to the front of the drum during mixing. The front of the drum is located behind the driver’s seat (Fig. 1). Along the length of the drum, a blade is attached perpendicular to the side of the drum, making a relative angle, *θ*, with the axis of the drum. This angle determines the pitch of the spiral made by the blades and it ranges from 55° to 70°. The average value of the cotangent of the angle can be calculated as follows:

$$\text{cotan}\theta =\frac{\frac{1}{\text{tan}55}+\frac{1}{\text{tan}70}}{2}=0.532.$$ (2)

This value is used to calculate the velocity of the concrete, *V*_c–m_, inside the drum as it moves forward due to combined effect of the blades and the drum rotation, as shown in Eq. (3).

$$V_{c-m}=V_{t-m}\cdot \text{cotan}\theta =2.1\cdot 0.532=1.11m/s$$ (3)

An approximate value of the shear rate in the concrete, ${\dot{\gamma }}_{c}$, can be calculated using Eq. (4):

$${\dot{\gamma }}_{c}=\frac{\left(\frac{\delta }{t}\right)}{y}=\frac{\text{relative velocity}}{\text{thickness of the sheared concrete}}$$ (4)

where:

*δ*: side length of the element of concrete considered (Fig. 2a)
*y*: the displacement during the time interval *t*
*t*: time required for the displacement of the element of concrete considered

For example, if *δ* is 0.06 m, with a *V*_c–m_ of 1.11 m/s, then the interval *t* is equal to 0.06/1.11 or 0.054 s and the shear rate:

$${\dot{\gamma }}_{c}=\frac{\left(\frac{0.06}{0.054}\right)}{0.06}=18.51s^{-1}$$ (5)

There are other regions of higher shear rates between the nearly static material along the blade at the shell and these flowing elements. However, as Ref. [12] explains, it can be estimated that the maximum shear rate applied to concrete in the drum does not exceed 30 s^−1^.

#### 2.3.2 ICAR Rheometer

**Fig. 3:**
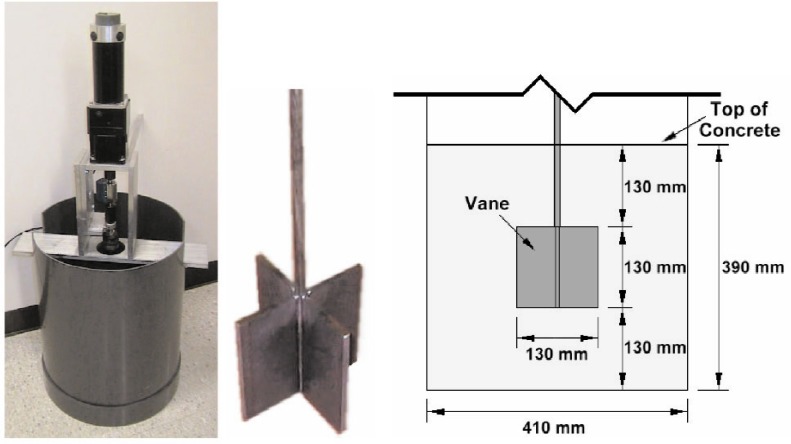
View of the ICAR rheometer prototype, vane, and principal dimensions.

The ICAR rheometer [13,14], shown in prototype form in Fig. 3, is a portable rheometer for fresh concrete. The device utilizes a four-bladed vane that is immersed into the concrete sample and rotated at a series of fixed speeds. The entire rheometer is approximately the size of a hand-drill and can be either operated by hand or secured into a fixed position above a standard container.

For each concrete mixture, the ICAR rheometer was used to perform a stress growth test and to measure a flow curve. In the stress growth test, the vane was rotated at a constant speed of 0.16 rad/s (0.025 rps) while the torque was measured. The peak torque was recorded as an approximation of the yield stress as discussed in Ref. [15,16,17]. After this peak torque was reached, the flow curve was then measured. The vane was first rotated at a speed of 6.3 rad/s (1.0 rps) for a breakdown period of 25 s. Torque measurements were then recorded for five speeds ranging in descending order from 6.3 rad/s to 1.3 rad/s (1.0 rps to 0.2 rps). The resulting data were analyzed based on the Bingham model, whereby a straight line was fit to the plot of torque, *T*(N·m), versus rotation speed, *N* (rad/s):

T=Y+VN. (6)

The intercept, *Y*(N·m), and the slope, *V*(N·m·s), of this line were considered to be related to yield stress and plastic viscosity, respectively. Due to the geometry of this rheometer, it is not possible to determine the shear rate analytically in fundamental units [13].

The concrete was placed in a 410 mm diameter container and filled to a height of 390 mm, as shown in Fig. 3. The vane, which measured 130 mm in diameter and 130 mm in height, was positioned in the center of the concrete sample, resulting in a gap size of 140 mm between the vane and the sidewalls and a gap of 130 mm above and below the vane.

### 2.4 Testing Procedure

**Fig. 4:**
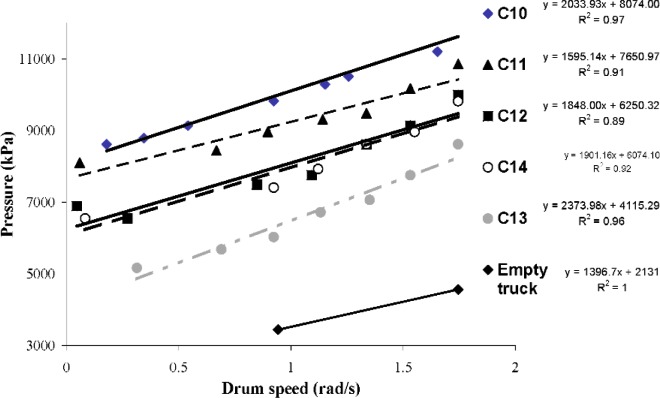
Flow curves obtained with the truck mixer (empty truck, C10 to C14 mixtures).

The truck with an empty drum was tested initially by measuring the oil pressure versus the rotation speed. This procedure, which was done only once, is shown in Fig. 4. It was not possible to measure pressure below 3.44 MPa (500 psi), therefore, preventing the measurement of more than two data points. This curve should be used to correct the measurements done with a concrete loaded truck in order to measure only the influence of the concrete on the torque measured. In this case, the curve obtained is not precise enough to be used and therefore, the values reported here are not corrected.

The concrete was mixed in the central plant mixer for 10 min and then transferred to the concrete truck mixer. For the first set of tests (C10 to C14), the truck was filled to 50 % of its maximum capacity, and then more concrete was added on top for the second set (C20 to C23) to reach 100 % of its maximum capacity. The purpose of this sequence was to determine the influence of the load on the results. Since the concrete was setting and therefore the second series was quite different from the first, it was not possible to really detect the influence of load volume on the results.

After loading, the truck was moved to the laboratory location: therefore, the first laboratory test on the concrete began 30 min after the first contact of water and cement. The truck drum turned about 100 revolutions during the transport of the concrete between the central plant and the laboratory. For each mixture, a small volume of concrete was discharged from the truck for testing with the ICAR rheometer and the slump test. These tests were conducted concurrently with the measurements from the truck. The temperature of the concrete was also recorded.

After a set of measurements, the concrete was modified by incorporating an admixture or adding water (see Table 1). The concrete was then remixed in the truck and tests were repeated. Five mixtures were prepared for the first set of tests (C10 to C14), and four test mixtures were prepared for the second set of tests (C20 to C23).

To use the truck as a rheometer, the highest speed of the drum (1.74 rad/s or 16.66 rpm) was maintained for 10 revolutions while the oil pressure was recorded. The speed of the drum was then reduced in increments of 0.21 rad/s (2 rpm). The oil pressure and speed were recorded at each increment of speed. These measurements produced the curve of oil pressure (related to the torque) vs. rotational speed used to calculate the yield stress and plastic viscosity.

## 3. Results and Discussion

### 3.1 Concrete Test Data

**Fig. 5:**
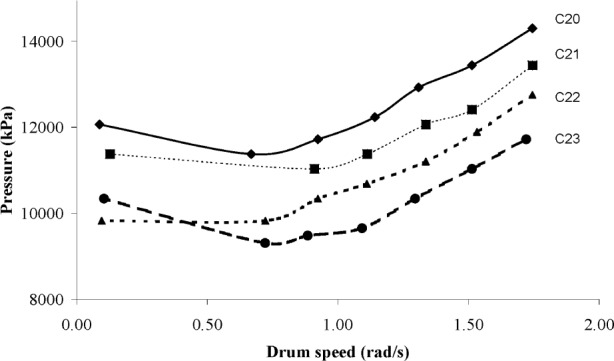
Flow curves obtained with the truck mixer (C20 to C23 mixtures).

**Fig. 6:**
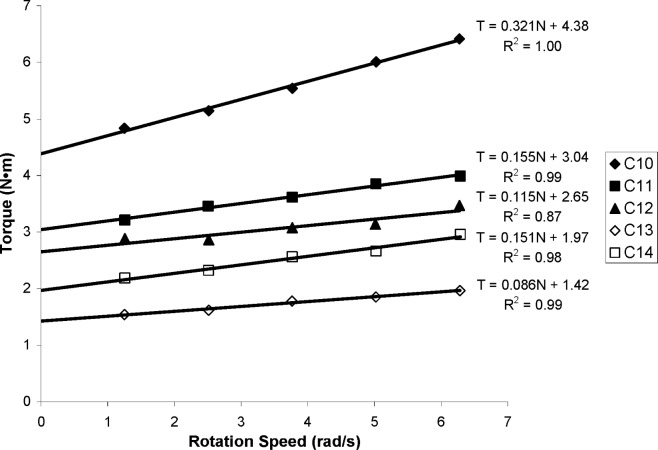
Flow curves obtained with ICAR rheometer for Series 1 (C10 to C14 mixtures).

**Fig. 7:**
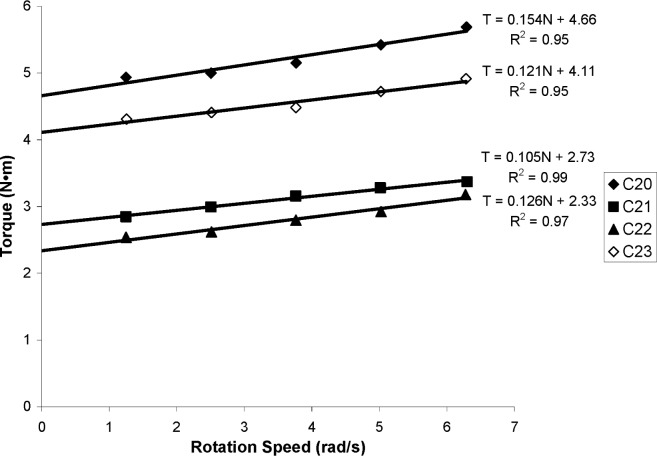
Flow curves obtained with ICAR rheometer for Series 2 (C20 to C23 mixtures).

The fresh concrete measurements are summarized in Table 2. The flow curves obtained from truck measurements are shown in Figs. 4 and 5, while the flow curves obtained from the ICAR rheometer are shown in Figs. 6 and 7. The values of the yield stress and plastic viscosity are deduced from these flow curves, as described by the Bingham model, by calculating the intercept of the linear fit for the yield stress and the slope as the plastic viscosity.

**Fig. 8:**
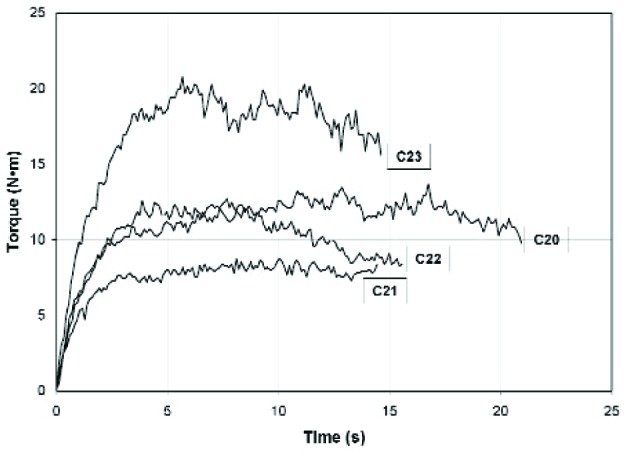
Typical ICAR rheometer stress growth test results for selected mixtures.

In general, the linear fit of the data for the ICAR rheometer is excellent, as indicated by an average *R*^2^ value of 0.965 for the nine flow curves. Typical stress growth test plots of torque versus time from the ICAR rheometer are shown in Fig. 8 for four selected concrete mixtures. The plots are characterized by an initial linear, elastic response followed by a nonlinear, viscoelastic response up to the peak torque (see Refs. [15,16,17] for more discussion of stress growth tests). After the peak torque is reached, viscous flow occurs and the torque begins to decay gradually. The variation in torque readings is typical for stress growth tests of concrete due to the wide range of aggregate sizes present.

### 3.2 Evolution of Plastic Viscosity

**Fig. 9:**
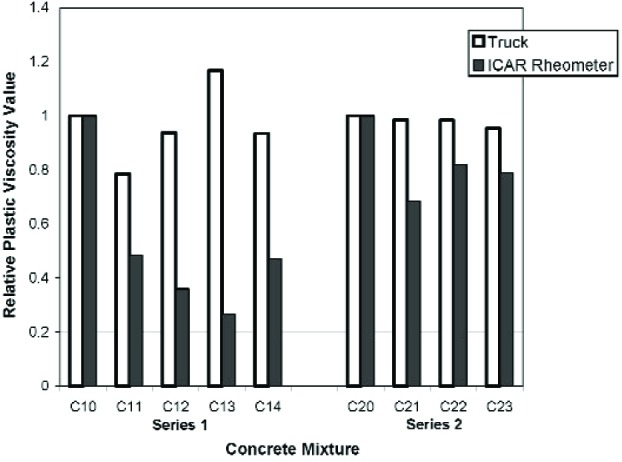
Relative plastic viscosity, as defined by the ratio of the measured plastic viscosity with the control mixture plastic viscosity, recorded by the mixing truck and ICAR rheometer. The control mixtures are C10 and C20. As the results shown are from one measurement, it is not possible to show the uncertainty.

In Fig. 9, the relative viscosity is shown for all the tests performed. The relative viscosity is defined as the ratio of the measured viscosity for a given test to the viscosity measured for the respective control mix C10 or C20. The addition of the first dosage of HRWRA to the control mix C10 resulted in a decrease in viscosity as measured by both the truck and the ICAR rheometer. Further additions of HRWRA to the same mix, however, produced different results from the two devices. The ICAR rheometer indicated that further additions of HRWRA resulted in further reductions in viscosity while truck measurements indicated that the viscosity began to increase. The use of a viscosity-modifying admixture (VMA), a product intended to increase viscosity, for mix C14 resulted in an increase in viscosity as recorded by the ICAR rheometer but a decrease in viscosity as recorded by the truck.

In the second series of mixtures, the addition of the first dosage of HRWRA resulted in a decrease in viscosity as recorded by both the truck and ICAR rheometer. Like the first series, the magnitude of the decrease in relative viscosity was greater for the ICAR rheometer than for the truck measurement. For the final two mixtures, the setting of the concrete began to dominate the rheology. The addition of a second dosage of HRWRA for mix C22 resulted in an increase in viscosity as measured by the ICAR rheometer, while the truck rheometer recorded a value of viscosity that was essentially the same as mix C21. Finally, the addition of water for mix C23 resulted in a decrease in viscosity as recorded by both the truck and the ICAR rheometer.

**Fig. 10:**
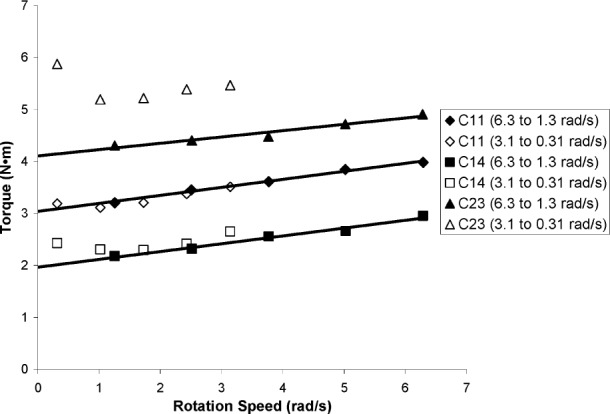
Illustration of slope of the flow curves for selected ICAR rheometer measurements.

In addition to the flow curve measurements indicated above, a second flow curve was measured with the ICAR rheometer over a lower range of rotation speeds (from 3.1 rad/s to 0.31 rad/s). The concrete sample was remixed by hand between each flow curve measurement; therefore, the second flow curve was measured approximately 3 min to 4 min after the first flow curve. In the first measurements (C10 to C14), the second series of flow curve points simply extended the original flow curve closer to the origin. However, as the day progressed and the concrete began to set, torque readings at low rotation speeds suggested a negative slope of the curve at low shear rates, as illustrated in Fig. 10. Since the plastic viscosity is defined as the slope, it would imply that this procedure resulted in the calculation of a negative viscosity, which is not physically possible. The final mixture exhibited not only a negative slope, but an upward shift of the entire curve. This same negative slope was evident in the truck measurements shown for mixture C12 in Fig. 4. When the rotation speed is sufficiently slow, the microstructure of the concrete is able to reform—that is, the cement particles are able to agglomerate during the shearing process resulting in an increase in torque. This phenomenon of a negative slope of the shear stress-shear rate curve was also observed for cement paste rheological tests where the value of the plastic viscosity was strongly dependent on the shear rate applied and the degree of hydration of the cement paste. Further explanations are presented in [17].

**Fig. 11:**
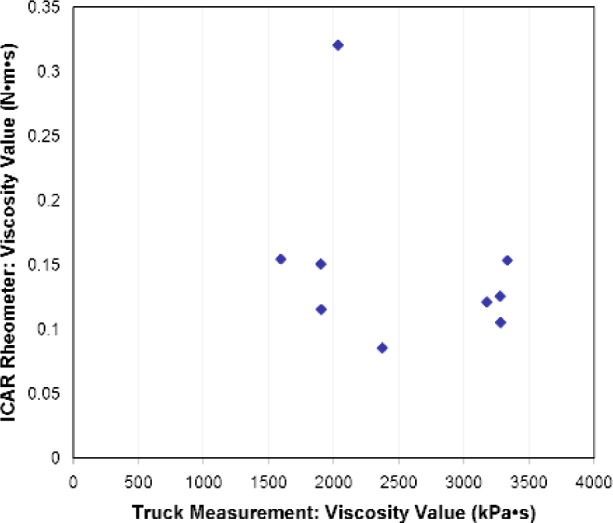
Correlation of viscosity value between ICAR rheometer and truck.

The correlations between the ICAR rheometer and truck measurements for the viscosity values are shown in Fig. 11. No apparent relationship exists for plastic viscosity. This disappointing result could be attributed to the lack of precision of the pressure gauge (± 0.34 MPa or 50 psi accuracy), especially at pressures less than 3.45 MPa (500 psi) where the lack of graduation below this lower value prevented measurements. Another possible reason for the discrepancy is the shear-thinning nature of concrete, resulting in a plastic viscosity that is shear rate dependent [17]. It is easy to state that the ICAR rheometer and the truck do not shear the concrete at the same shear rate, leading to different plastic viscosities. This does not explain the different trends in the relative viscosities. Nevertheless, the results show that the truck is able to sense differences between the mixes as the plastic viscosity value varies between 1500 kPa·s and 4000 kPa·s.

### 3.3 Evolution of Yield Stress

**Fig. 12:**
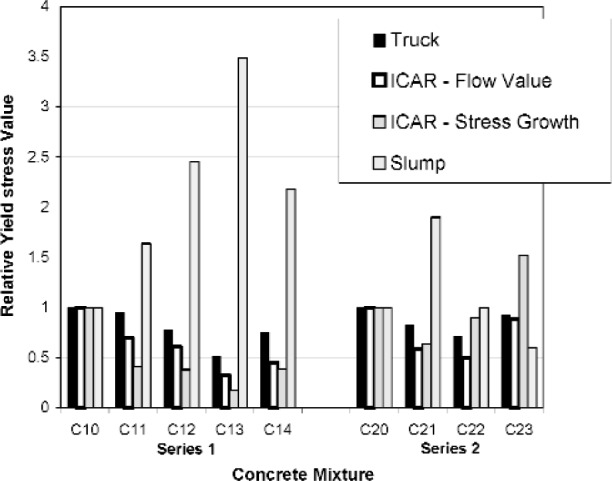
Relative yield or slump values, as defined by the ratio of the measured yield stress or slump with the control mixture yield stress or slump, as recorded by mixing truck, ICAR rheometer, and slump test. The control mixtures are C10 and C20.

**Fig. 13:**
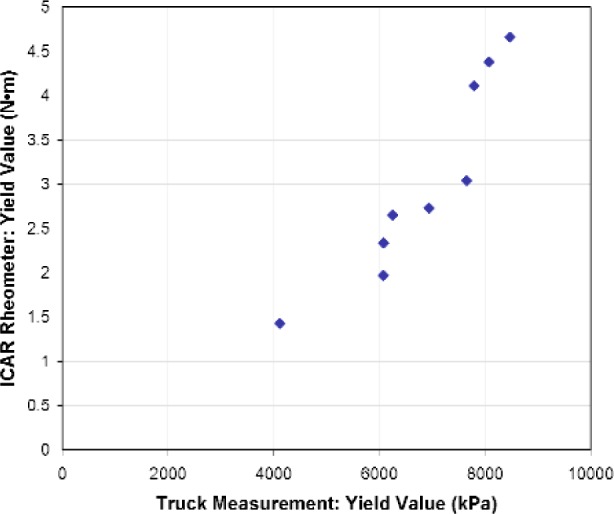
Correlation of yield value between ICAR rheometer and truck.

The relative variations in yield stress as recorded from the truck measurements, ICAR rheometer, and the slump test are shown in Fig. 12 and Fig. 13. The results between different tests were generally consistent. The addition of HRWRA for mix C11, C12, and C13 resulted in decreases in the yield values measured by both the ICAR rheometer and the truck. Many authors [18,19,20] have shown that concrete slump is inversely correlated to yield stress, because a high slump indicates a reduced resistance to flow as does a low yield stress. Indeed, the slump increased as the truck measurements and ICAR rheometer indicated a reduction in yield stress due to the addition of HRWRA. The use of a viscosity-modifying admixture for mix C14 resulted in increases in yield value as determined by both the truck and the ICAR rheometer and a decrease in slump as compared to mix C13. For the second mixture, the use of HRWRA again resulted in reductions in yield value as determined by the ICAR rheometer and the truck and an increase in slump. As with the viscosity values, the yield values were dominated by the setting of the concrete for the last two mixtures.

## 4. Conclusions

Based on the limited investigation (only one day of testing) of the feasibility of measuring rheological properties directly in a mixing truck without any modifications, it was determined that the mixing truck can be used as a tool to obtain flow curves of the mixed material, with the same procedure used with a concrete rheometer, and that the flow curves measured by the mixing truck were sensitive to changes in yield stress and plastic viscosity.

The comparison of the yield stress measured with the slump test, the ICAR rheometer, and the mixing truck showed a good correlation between the values measured. On the other hand, the plastic viscosity measured by the truck or the ICAR rheometer did not show a high correlation. The results of the ICAR rheometer measurements appeared to be more realistic than the truck measurements for changes in plastic viscosity due to addition of admixtures or water. This situation could be attributed to various factors. For instance, the truck measurements had several sources of uncertainty in the precision of the measurements of the drum rotation speed and torque. A more precise gauge for the torque is essential, as it is probably the largest source of uncertainty. An automated speed measurement would also be desirable, although it is probably not a major source of error in this study. Calibration methods are needed to obtain the results in fundamental units and to better compare the test results with other rheometers. A more accurate knowledge of the drum geometry might also lead to the calculation of the rheological parameters in fundamental units.

In summary, this preliminary study showed the feasibility of measuring the slope and intercept of the torque vs. speed plot from a mixing truck drum as a means of determining concrete workability. The yield stress measured with this method correlated well with the results of the slump test and the ICAR rheometer. More extensive studies are needed for a reliable measurement of the viscosity.

## Figures and Tables

**Table 1:** Mixture proportions^a^

		First concrete batch (50 % Capacity)					Second concrete batch (100 % Capacity)			
		C10	C11	C12	C13	C14	C20	C21	C22	C23
Gravel (oven dry)	kg/m^3^	1099.4	← Constant →				+640.4	← Constant →		
Sand (oven dry)	kg/m^3^	774.0	← Constant →				+1042.8	← Constant →		
Water (free and absorbed)	kg/m^3^	145.8	← Constant →				+157.0	← Constant →		
Cement	kg/m^3^	163.7	← Constant →				+222.5	← Constant →		
Slag	kg/m^3^	163.2	← Constant →				+223.1	← Constant →		
Set retarder	L/m^3^	0.656	← Constant →				+1.0	← Constant →		
HRWRA	L/m^3^	0	+1.4	+1.4	+0.6			+4.0	+4.0	
VMA	L/m^3^	0					+0.2				

Testing time	h	0.5	1.0	1.5	2.0	2.4	0.5	1.0	1.6	2.1
Temperature	°C	18	19.5	20	20	21	29.5	30	31	31
Slump	mm	70	110	170	240	150	60	120	60	40

a The water quantity calculations take into account the initial moisture contents of the aggregates.

**Table 2:** Fresh concrete measurements

		Truck Measurements		ICAR Rheometer		
Mixture designation	Slump (mm)	Yield stress value (kPa)	Plastic viscosity value (kPa·s)	Yield stress value (N·m)	Plastic viscosity value (N·m·s)	Stress growth Max torque (N·m)
Empty truck		2131	1397			
C10	70	8074	2034	4.38	0.321	24.26
C11	110	7651	1595	3.04	0.155	9.98
C12	170	6250	1848	2.65	0.115	9.26
C13	240	4115	2374	1.42	0.086	4.18
C14	150	6074	1901	1.97	0.151	9.42
C20	60	8472	3334	4.66	0.154	13.69
C21	120	6939	3282	2.73	0.105	8.77
C22	60	6080	3278	2.33	0.126	12.30
C23	40	7792	3177	4.11	0.121	20.77

## Footnotes

2 Information provided by the manufacturer without an uncertainty statement.
